# Supplementary material for: Elucidating a statistical learning brain network: Coordinate-based meta-analyses and functional connectivity profiles of artificial grammar learning in healthy adults
Source: Imaging Neurosci (Camb). 2024 Nov 7;2:imag-2-00355. doi: 10.1162/imag_a_00355 (PMC12290583; doi:10.1162/imag_a_00355)
Supplement: Supplementary Material [file imag_a_00355-supp.pdf]

Supplemental Materials

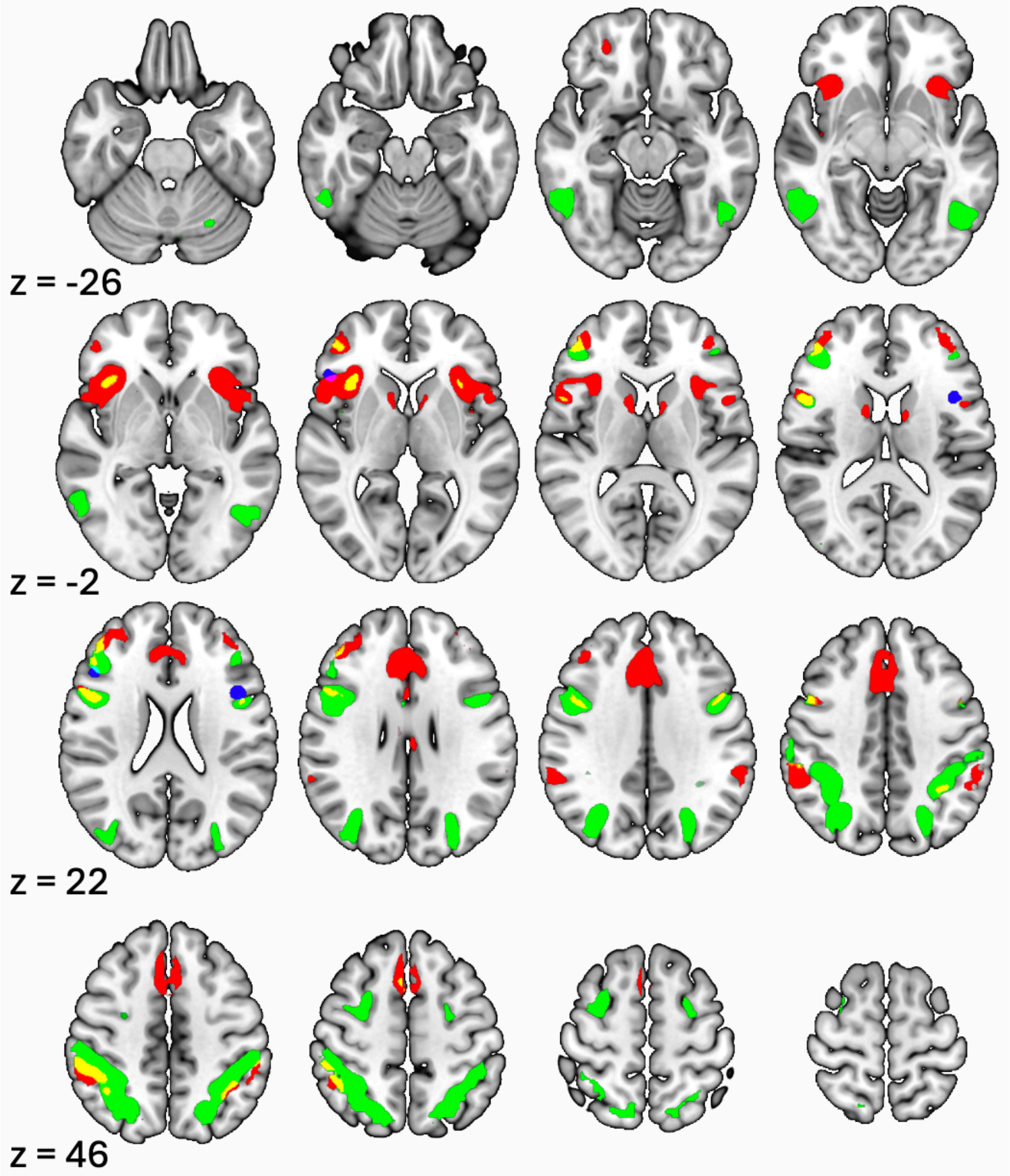

Supplemental Figure 1. The analyses identified language (blue), salience (red), and cognitive control (green) cliques that had unique as well as some overlapping spatial representations (yellow). Images generated in MRIcroGL (Rorden & Brett, 2000).

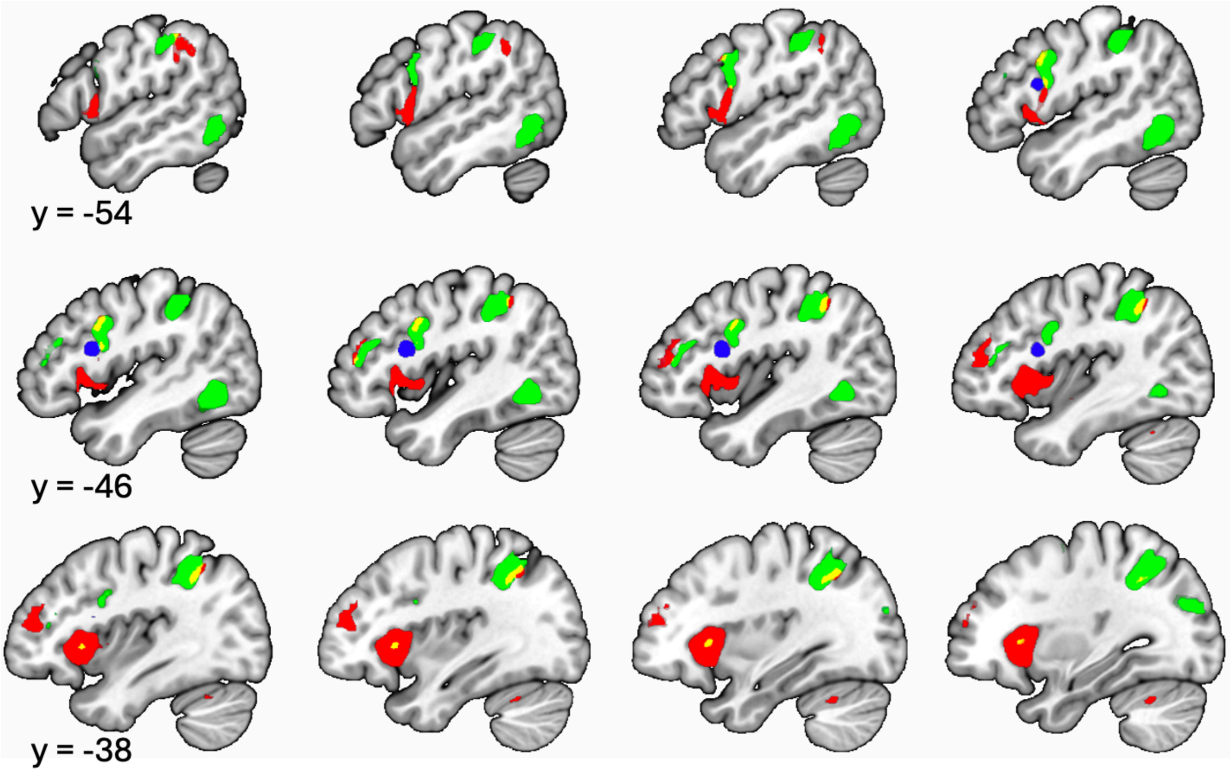

Supplemental Figure 2. All three cliques had representation in the left inferior frontal gyrus, though the only region uniquely identified in the hierarchical clustering was the opercular IFG in Clique 1 (middle row, blue). The ventral medial left IFG was associated with Clique 2 (red), with some overlap with Clique 3 (green). Images generated in MRIcroGL (Rorden & Brett, 2000).

ALE GROUP	CLUSTER	X	Y	Z	Volume (mm³)	ALE Max	Label
GRAMMATICAL							
	1	-46	8	14	6096	5.08	L Inferior Frontal Opercularis
		-46	*28*	*28*		*4.84*	*L Inferior Frontal Triangularis*
		-46	*2*	*30*		*4.15*	*L Precentral Gyrus*
	2	-30	22	-2	1536	6.45	L Insula
	3	32	-72	38	976	4.47	R Middle Occipital Gyrus
	4	34	24	0	888	4.63	R Insula
	5	-42	-28	10	832	4.55	L Superior Temporal Gyrus
UNGRAMMATICAL							
	1	-44	12	22	7504	6.56	L Inferior Frontal Opercularis
		-38	*20*	*0*		*5.78*	*L Insula*
	2	48	26	18	6560	6.21	R Inferior Frontal Triangularis
		48	*18*	*42*		*4.88*	*R Middle Frontal Gyrus*
		46	*32*	*-4*		*3.33*	*R Inferior Frontal Orbitalis*
	3	6	26	32	3224	5.31	R Middle Cingulate Gyrus
		0	*24*	*52*	*3224*	*4.40*	*L Supplemental Motor Area*
	4	36	22	-4	1856	7.00	R Insula
CONJUNCTION							
	1	-44	10	18	1856	4.74	L Inferior Frontal Opercularis
		-44	*26*	*22*		*3.74*	*L Inferior Frontal Triangularis*
	2	-34	20	-2	520	5.02	L Insula

Supplemental Table 1. Results for the grammatical and ungrammatical ALE contrasts and their conjunction.

CLUSTER	VOXELS	X	Y	Z	MACROANATOMY	CYTOARCHITECTURE
**1**	1393	50	26	4	34% IFG pars triangularis; 6% IFG Opercularis	45/44/OP8/OP9
		48	*24*	*22*	20% MFG, 18% IFG pars Opercularis	45/44
		58	*16*	*20*	62% IFG pars Opercularis; 10% Precentral Gyrus	44/45
		50	*40*	*0*	70% Frontal Pole; 12% IFG triangularis	45
		50	*8*	*42*	41% MFG, 30% Precentral Gyrus	44
		50	*10*	*38*	40% MFG, 28% Precentral	44
**2**	553	-40	10	18	41% IFG Opercularis, 10% MFG	44
**3**	113	-38	22	-2	36% Insula, 25% Frontal Orbital Cortex	Id7
**4**	10	-50	36	8	43% IFG triangularis; 31% Frontal Pole	45
**5**	10	-44	28	20	44% IFG triangularis; 25% MFG	45

Supplemental Table 2. Clusters making up Clique 1, associated with language terms in NeuroSynth. Cluster identification and labeling from the Anatomy Toolbox (Eickhoff et al., 2005, 2006; Zilles & Amunts, 2010). IFG = inferior frontal gyrus, MFG = middle frontal gyrus.

CLUSTER	VOXELS	X	Y	Z	MACROANATOMY	CYTOARCHITECTURE
**1**	1492	6	24	34	50% Paracingulate Gyrus; 36% Anterior Cingulate Gyrus	
		4	22	50	60% SFG, 26% paracingulate gyrus	
		-2	22	48	35% SFG, 30% paracingulate gyrus	
		0	18	46	51% Paracingulate Gyrus, 4% SFG	
		2	22	42	71% Paracigulate Gyrus, 9% Anterior Cingulate	
		-2	14	52	36% Paracingulate Gyrus, 24% SFG	6mr/preSMA
		-2	28	44	34% SFG, 31% Parcing	
		-8	28	28	44% Paracingulate Gyrus, 39% Cing (anterior)	
		0	10	28	87% Cingulate Gyrus (anterior)	
		-2	32	50	73% SFG	
**2**	1373	36	22	0	47% Insula, 16% Frontal Operculum	id7, 45
		54	12	8	58% IFG; 18% Precentral Gyrus	44
		52	12	22	48% IFG pars opercularis; 25% Precentral gyrus	44
		44	6	36	33% MFG, 30% Precentral Gyrus	44
		50	8	38	42% Precentral Gyrus, 34% MFG	44
		46	12	32	28% MFG, 21% Precentral Gyrus	44
**3**	907	-34	24	-2	38% Insula; 34% Frontal Orbital Cortex	id7
**4**	692	44	50	8	86% Frontal Pole	
		44	38	30	61% Frontal Pole; 29% MFG	
		40	52	20	74% Frontal Pole	
		48	44	2	90% Frontal Pole; 2% IFG p traingularis	
		42	48	20	85% Frontal Pole	
		32	52	20	83% Frontal Pole	
		32	50	28	86% Frontal Pole	
		34	46	30	85% Frontal Pole	
		48	40	16	71% Frontal Pole; 11% MFG	
		46	44	-6	91% Frontal Pole	
**5**	601	56	-42	48	66% Supramarginal Gyrus; 20% Angular Gyrus	PFm (IPL), PF(IPD); hIP2(IPS)
**6**	244	-58	-40	44	44% Supramarginal Gyrus (44% anterior division, 34% posterior division)	PF (IPL); hIP2(IPS); PFm(IPL)
**7**	206	-38	50	16	85% Frontal Pole	
**8**	108	10	10	6	91% Right Caudate	
**9**	95	-38	-50	42	25% Supramarginal Gyrus (posterior division); 21% Angular Gyrus	hIP1(IPS); hIP2(IPS), hIP3(IPS), 7PC(SPL)
**10**	60	-8	4	6	53% Left Caudate	
**11**	47	-48	8	38	33% Middle Frontal Gyrus; 21% Precentral Gyrus	44

**12**	42	-32	-56	-32	78% Cerebellum VI; 20% Left Crus I	
**13**	34	26	48	-12	58% Frontal Pole	Fo3
**14**	14	42	-6	-12	61% Planum Polare; 15% Insular Cortex	Id1
**15**	14	-4	-20	28	43% Posterior Cingulate Gyrus; 11% Anterior Cingulate Gyrus	
**16**	12	16	8	68	56% Superior Frontal Gyrus	6d2
**17**	10	6	-12	30	23% Cingulate Gyrus (anterior); 11% Cingulate Gyrus (posterior)	33

Supplemental Table 3. Supplemental Table 2. Clusters making up Clique 2, associated with salience terms in NeuroSynth. Cluster identification and labeling from the Anatomy Toolbox (Eickhoff et al., 2005, 2006; Zilles & Amunts, 2010). SFG = superior frontal gyrus, MFG = middle frontal gyrus; mr6/SMA = supplementary motor area, id = Insular dysgranular area, PF = PF areas of the left supramarginal area including PFcm, PFm, PFt, and PFop; IPL = inferior parietal lobule, IPS = inferior parietal sulcus; hIP2 = area of the left inferior parietal sulcus; PC = precuneus; Fo = frontal operculum.

CLUSTER	VOXELS	X	Y	Z	MACROANATOMY	CYTOARCHITECTURE
**1**	2566	30	-66	38	40% lateral occipital cortex; 13% SMG	hIP5, hIP6, hIP8
		36	-50	46	37% SPL, 26% Angular	hIP3, hIP2, hIP1
		50	-34	54	30% SMG (anterior), 26% SMG (posterior)	1, 2, PFt(IPL), PF(IPL), PFm(IPL)
		28	-66	52	60% Lateral Occipital Cortex	7A, hIP3(IPS)
		56	-28	50	62% SMG (anterior), 8% Postcentral	PFt(IPL), PF(IPL), 2, hIP2(IPS), 1
		42	-46	58	57% SPL, 10% Angular	hIP3(IPS), 7PC(SPL), PGa(IPL)
		18	-70	54	52% Lateral Occipital Cortex (superior)	7P, 7A
		12	-70	58	58% Lateral Occipital Cortex (superior), Precuneus	7P, 7A
		12	-68	62	59% Lateral Occipital Cortex(superior), Precuneus	7P, 7A
		8	-66	62	34% Lateral Occipital Cortex, 31% Precuneus	7P, 7A
**2**	1696	-26	-72	34	38% Lateral Occipital cortex; 19% SPL	hIP5, hIP8
		-24	-70	48	58% Lateral Occipital Cortex (superior)	hIP6, 7A, hIP8
		-24	-66	38	54% Lateral Occipital Cortex, Precuneus	hIP8, hIP3, hIP5, hIP6
		-32	-54	48	42% SPL, 11% Angular	hIP3, 7PC, hIP1, 7A
		-40	-48	50	41% SPL, 22% SMG	hIP3, 7PC, hIP1, hIP2
		-38	-46	44	33% SPL, 32% SMG	hIP1, hIP3, hIP2, 7PC
		-42	-46	52	39% SPL, 26% SMG	hIP3, 7PC, hIP1, 5L, hIP2
		-46	-40	48	23% SMG (posterior); 21% SMG (anterior)	2, PFt(IPL)
		-50	-36	50	38% SMG (anterior); 18% Postcentral Gyrus	2, PFt(IPL), 1
		-16	-74	52	70% Lateral Occipital Cortex, 3% Precuneus	hIP8, 7A, 7P
**3**	1211	46	4	30	25% Precentral, 24% Frontal Pole	44
**4**	616	54	-56	-12	71% ITG, 8% MTG (temporooccipital)	
**5**	453	-50	-64	-10	49% Lateral Occipital Cortex; 29% ITG	FG4, FG2
**6**	327	30	2	60	37% MFG, 24% SFG	6d3
**7**	298	-48	6	36	35% MFG; 34% Precentral	44
**8**	114	-26	0	58	33% SFG, 27% MFG	6d3, 6d2
**9**	105	-46	32	24	70% MFG, 12% Frontal Pole	45
**10**	104	38	22	0	33% Frontal Operculum; 29% Insula	Id7, 45, OP9, OP8
**11**	48	-26	-72	-50	85% Cerebellum VIIb, 9% Left Crus II	
**12**	26	-26	-66	-26	96% Cerebellum VI; 3% Crus I	
**13**	25	4	18	50	51% Paracingulate Cortex; 32% SFG	6mr/pre-SMA
**14**	19	-36	18	2	62% Insula; 16% Frontal Operculum	Id7, OP8, 44
**15**	10	26	-72	-50	78% Cerebellum Iib, 15% Crus II	
**16**	10	2	4	30	87% Cingulate Cortex (anterior)	33

Supplemental Table 4. Clusters making up Clique 3, associated with cognitive control terms in NeuroSynth. Cluster identification and labeling from the Anatomy Toolbox (Eickhoff et al., 2005, 2006; Zilles & Amunts, 2010). SMG = supramarginal gyrus, SPL = superior parietal lobule, ITG =

inferior temporal gyurs, MTG = middle temporal gyurs, SFG = superior frontal gyrus, MFG = middle frontal gyrus

FEATURE	CLIQUE 1	CLIQUE 2	CLIQUE 3
ABSTRACT	0.17748751	0.02031971	0.17628204
ABUSE	-0.0918497	-0.0340203	-0.1271657
ACCURACY	0.0846616	0.12605231	0.09021144
ACCURATE	0.06828813	0.03557864	0.09969926
ACCURATELY	0.08145894	0.07062794	0.05823978
ACHIEVE	-0.0373396	-0.0336285	-0.0029944
ACHIEVED	-0.0804157	-0.1073192	-0.0663505
ACOUSTIC	0.0339713	-0.0299751	-0.0902833
ACT	0.00098255	-0.0038895	-0.0158629
ACTION	0.12649455	0.0539338	0.26653555
ACTION OBSERVATION	0.06148967	-0.022127	0.2170404
ACTIONS	0.0992535	-0.0153651	0.21347198
ACTIVITIES	-0.0297307	-0.0297382	0.02866528
ACUTE	-0.1343907	-0.0432464	-0.1855745
ADAPTATION	0.02122583	0.00132972	0.11366213
ADAPTED	0.0096768	0.04488608	0.01859152
ADAPTIVE	0.07685508	0.16558552	0
ADD	-0.0517754	-0.0019695	0.01061507
ADDICTION	-0.1200386	-0.0285448	-0.1518885
ADDITION	0.08947953	0.08592672	0.10362291
ADHD	-0.0689445	-0.0283938	-0.1348892
ADOLESCENCE	-0.0609287	-0.0245583	-0.0434199
ADOLESCENT	-0.0471575	0.00104855	-0.1452948
ADOLESCENTS	-0.1074343	-0.0590134	-0.165352
ADULT	-0.0134572	-0.0651341	-0.1118094
ADULTHOOD	-0.0403566	-0.0766378	-0.1204609
ADULTS	0.03027956	0.03681665	0.00297147
AFFECT	0	0	-0.1307146
AFFECTIVE	-0.0044174	0.01027694	-0.243383
AGING	0	0.01048267	0.00361858
ALCOHOL	-0.0682315	-0.0312162	-0.0905808
ALZHEIMER	-0.1574023	-0.174837	-0.1644208
ALZHEIMER DISEASE	-0.1560028	-0.1723263	-0.1577206
AMBIGUOUS	0.13821311	0.02641948	0
AMNESTIC	-0.0636392	-0.0758419	-0.0935899
ANGER	-0.0580625	-0.0920668	-0.1624266
ANGRY	0	-0.0847477	-0.1348352
ANTICIPATION	-0.0810381	0.13691861	-0.0720204
ANTICIPATORY	-0.0668854	0.04682427	-0.0390057
ANXIETY	-0.140435	-0.049028	-0.2121016
ANXIETY DISORDERS	-0.1123703	-0.0483774	-0.1354589
APHASIA	0.08562149	-0.0266921	-0.0262079

**APPRAISAL**	-0.0584513	0	-0.0998538
**ARITHMETIC**	0.12048007	0.13991101	**0.31564795**
**AROUSAL**	-0.078325	-0.01862	-0.1396284
**ARTICULATORY**	0.14747941	0.1085229	0.06051318
**ASD**	0.01524249	-0.0322125	-0.0675823
**ATROPHY**	-0.1515331	-0.1774178	-0.2006015
**ATTEMPT**	0.09439089	0.09812689	0.05576026
**ATTEMPTED**	0.04128342	0.01771116	0.01033765
**ATTEND**	0.06488673	0.02380362	0.14610183
**ATTENDED**	0.03309052	-0.0484765	0.17245299
**ATTENDING**	0.05035889	0.02244777	0.14167621
**ATTENTION**	0.06978283	0.12646302	**0.37395251**
**ATTENTION DEFICIT**	-0.0654737	-0.0244716	-0.1250754
**ATTENTION NETWORK**	0.02762754	0.0729104	0.25914074
**ATTENTION TASK**	0.0294059	0.08429311	0.19280979
**ATTENTIONAL**	0.12650065	0.18836217	**0.39837797**
**ATTENTIONAL CONTROL**	0.135859	0.16520491	0.22531363
**ATYPICAL**	0.01511591	0	-0.0252721
**AUDIOVISUAL**	0.04576326	-0.0715222	-0.0147827
**AUDITORY**	0.08268947	0	-0.0503531
**AUDITORY STIMULI**	0.079371	0.03001678	0
**AUDITORY VISUAL**	0.08435659	-0.034899	0
**AUTISM**	-0.0343255	-0.1057647	-0.1032264
**AUTISM SPECTRUM**	-0.0054358	-0.0717569	-0.0770121
**AUTOBIOGRAPHICAL**	-0.0613529	-0.120609	-0.174502
**AUTOBIOGRAPHICAL MEMORY**	-0.0510745	-0.1191511	-0.1507112
**AUTOMATIC**	-0.0077529	-0.0314756	0.06778852
**AUTOMATICALLY**	-0.00084	-0.0558187	0.04088401
**AUTONOMIC**	-0.0740717	0.08041948	-0.0666128
**AVERSIVE**	-0.0416909	0.08019076	-0.1283419
**AVOID**	-0.026986	0.02452614	-0.0547943
**AVOIDANCE**	-0.0548954	0.01279837	-0.082697
**AWARENESS**	-0.0142455	0.04518585	-0.0749807
**BALANCE**	-0.0305598	0.0114598	-0.0605432
**BEHAVIOR**	-0.0118465	0.18405926	-0.0487756
**BEHAVIORS**	-0.0789291	-0.0157691	-0.1526411
**BEHAVIOUR**	0.05743206	0.03530019	-0.0258826
**BELIEF**	0.02075686	-0.0174683	-0.0510264
**BELIEFS**	0.03252945	-0.0186875	-0.1080784
**BELIEVED**	0.01370873	0.05441165	-0.0077701
**BILINGUALS**	0.21534917	0.09040369	0.0536226
**BIPOLAR**	-0.0185131	-0.0319977	-0.1766064
**BIPOLAR DISORDER**	-0.0187619	-0.0118516	-0.1571246
**BLIND**	-0.1337558	-0.1231295	-0.0724668

**BODILY**	0.00763508	0.02561836	-0.0186713
**BODY**	-0.0614862	-0.0704544	0.12384449
**CALCULATED**	-0.1283798	-0.0879861	-0.1567219
**CALCULATION**	0.13949648	0.19486251	**0.4050659**
**CAPACITY**	0.13075954	0.12552246	0.17769882
**CATEGORICAL**	0.02666577	-0.0208676	0.09412242
**CATEGORIES**	0	-0.0928409	0.05073892
**CATEGORIZATION**	0.07510249	0.06160531	0.12002989
**CATEGORY**	0.03543827	-0.0386831	0.05046617
**CHALLENGE**	-0.0081202	-0.0064556	-0.0648938
**CHALLENGES**	0	-0.0064322	0
**CHALLENGING**	0	0.00747068	0.04191287
**CHANGE**	-0.1106169	-0.0700736	-0.029587
**CHANGED**	-0.0530174	0	0.0121814
**CHANGING**	0.12001527	0.16972127	0.12515657
**CHARACTERS**	0.18347669	0.06205079	0.25663843
**CHILDREN**	0.03521479	-0.0173549	-0.0751886
**CHINESE**	0.22309199	0.10907773	0.19671567
**CHOICE**	0.02106907	0.17352313	0.02576433
**CHOICES**	0	0.14804602	-0.0342094
**CHOOSE**	0.05917883	0.18429091	0.01230219
**CHOSEN**	-0.0448475	0	0.04243154
**CHRONIC**	-0.1634708	-0.0799729	-0.2060188
**CHRONIC PAIN**	-0.1404318	0.00691905	-0.1070255
**CLASSICAL**	0.00747658	0.00167637	-0.0428763
**CLASSIFICATION**	-0.0504299	-0.072256	-0.066575
**CLASSIFIED**	-0.0324246	-0.0181066	0.0134033
**CLINICAL**	-0.2480931	-0.1889825	-0.3006435
**CLINICALLY**	-0.0712165	-0.0394149	-0.1220484
**CLIPS**	0.06456041	-0.0383554	0.07519583
**COGNITION**	0.04433951	-0.0187434	-0.0167917
**COGNITIVE**	0.17564987	0.24377584	0.12719103
**COGNITIVE CONTROL**	0.2175587	**0.33601983**	0.16034897
**COGNITIVE DEFICITS**	0.02989403	0.06206432	0.08330035
**COGNITIVE EMOTIONAL**	-0.0163844	0.00116337	-0.0906438
**COGNITIVE FUNCTION**	0.00681673	0.06124462	0.04796724
**COGNITIVE FUNCTIONS**	0.07204634	0.10482424	0.06848074
**COGNITIVE IMPAIRMENT**	-0.0834297	-0.0935601	-0.0860491
**COGNITIVE PERFORMANCE**	-0.0408245	0	0
**COGNITIVE PROCESSES**	0.13447683	0.20384416	0.12006953
**COGNITIVE TASK**	-0.0944147	-0.0291832	0.04704447
**COGNITIVE TASKS**	-0.0686195	0	0.04859927
**COGNITIVELY**	0	0.05068361	0
**COHERENCE**	-0.0313391	-0.0640289	-0.0752046

**COHERENT**	0	0	0.04892014
**COLOR**	0.11050351	0.09008664	**0.33718568**
**COMMUNICATION**	0.0695866	-0.0857644	-0.0735331
**COMPARE**	-0.0058929	0	0.04396625
**COMPARING**	0.01354656	0	-0.0205095
**COMPARISONS**	-0.0757083	-0.045419	-0.0603682
**COMPENSATE**	-0.0425139	0	0.01034486
**COMPENSATION**	0.03704852	0.05924041	0.05897578
**COMPENSATORY**	0.05756703	0.04205452	0.06063296
**COMPETING**	0.17099399	0.19368179	0.07963901
**COMPETITION**	0.14473851	0.14569017	0.13281956
**COMPLEX**	-0.0479308	-0.0887017	0.07605768
**COMPLEXITY**	0.1563444	0.09091715	0.08922195
**COMPREHENSION**	0.24789266	0	-0.0799604
**COMPULSIVE**	-0.047053	0	-0.1135024
**COMPULSIVE DISORDER**	-0.0500714	-0.0041573	-0.1080404
**COMPUTATION**	0	-0.015121	0.05739688
**COMPUTATIONAL**	-0.1007075	-0.0653364	-0.0098153
**COMPUTED**	-0.1156558	-0.0747931	-0.0993511
**CONCENTRATION**	-0.0481786	0	-0.0854906
**CONCEPT**	0	-0.0473229	-0.0137754
**CONCEPTS**	0.08489943	-0.0096516	0.07045436
**CONCEPTUAL**	0.15566426	-0.0396774	0.07420823
**CONCRETE**	0.14536054	0	0.03043289
**CONDITIONED**	-0.1235754	0	-0.1814745
**CONDITIONING**	-0.113686	0	-0.1974712
**CONFIDENCE**	-0.0407708	-0.0479587	0.01094099
**CONFLICT**	0.1837211	**0.32739212**	0.14288365
**CONFLICTING**	0.19364158	0.17325382	0.13947555
**CONGRUENCY**	0.18385014	0.15289216	0.17277234
**CONGRUENT**	0.11610567	0.07496005	0.18992419
**CONGRUENT INCONGRUENT**	0.06236308	0.039805	0.02501306
**CONSCIOUS**	-0.0204471	0.0599962	-0.0065843
**CONSCIOUSNESS**	-0.0475259	-0.0239462	-0.0051583
**CONSOLIDATION**	-0.1099427	-0.1450071	-0.1268875
**CONSTRUCTION**	-0.0105131	-0.080023	-0.0371324
**CONSUMPTION**	-0.0890266	-0.0406378	-0.1037048
**CONTEXT**	0.12458389	0.08751672	-0.0505213
**CONTEXTS**	0.08579937	0.02702479	-0.03295
**CONTEXTUAL**	0.07214456	0.04709668	-0.026628
**CONTINGENT**	0.01560501	0.07261978	0.05851675
**CONTROL**	0.12713108	0.28219917	0.18155706
**CONTROL NETWORK**	0.11634195	0.19031372	0.0920968
**CONTROL PROCESSES**	0.20296678	0.29092262	0.18909774

**CONTROL TASK**	0.19413152	0.17259983	0.20016183
**COORDINATION**	-0.0452707	0	0.094878
**CORRECT**	0.17076503	0.25777808	0.13604072
**CORRECTLY**	0.06265448	0.06169602	0.11736995
**COST**	0.04392835	0.03191485	0.04913926
**COSTS**	0.06645815	0.06125275	0.15529457
**COVERT**	0.05511924	0.01215714	0.12957892
**CRAVING**	-0.103102	-0.0898228	-0.0969861
**CUE**	0.08502024	0.15284638	0.20248573
**CUED**	0.11884502	0.11692556	0.19588182
**CUES**	0.01743673	0.01621837	0.0834922
**DECISION**	0.12292757	0.21688517	0.03369217
**DECISION MAKING**	-0.0082172	0.1624349	-0.0389727
**DECISION TASK**	0.14891647	0.0480282	0.04193924
**DECISIONS**	0.06945487	0.14719693	-0.0051346
**DEFAULT**	-0.1733935	-0.1115842	-0.1779545
**DEFAULT MODE**	-0.1800354	-0.1135487	-0.1884531
**DEFAULT NETWORK**	-0.0987443	-0.0802573	-0.0966496
**DEFICIT HYPERACTIVITY**	-0.0712013	-0.0296651	-0.1231972
**DELAY**	-0.0265394	0.05934445	0.07054025
**DELAYED**	0.03315499	0.11905895	0.14896983
**DEMAND**	0.12138179	0.10593081	0.20761398
**DEMANDING**	0.07373305	0.1320574	0.19525391
**DEMANDS**	**0.35482444**	**0.31495799**	**0.34238158**
**DEMENTIA**	-0.0981325	-0.1396621	-0.1556406
**DEPRESSED**	-0.0895351	-0.1180007	-0.2479315
**DEPRESSION**	-0.1684239	-0.172866	-0.3079248
**DEPRESSIVE**	-0.1665909	-0.1821472	-0.274503
**DEPRESSIVE DISORDER**	-0.1272256	-0.1394578	-0.228721
**DETECT**	-0.0223234	0	-0.0415834
**DETECTED**	-0.1117526	-0.0423108	-0.0394656
**DETECTING**	0.10650935	0.1302115	0.1204918
**DETECTION**	0.02277365	0.07011581	0.11764031
**DETECTION TASK**	0.02330366	0	0.1652504
**DEVELOPING**	-0.0536728	-0.091023	-0.1024518
**DEVELOPMENT**	-0.1017797	-0.1176251	-0.1201751
**DEVELOPMENTAL**	0.04042619	0	-0.0352419
**DIAGNOSED**	-0.1033193	-0.10167	-0.1614299
**DIAGNOSIS**	-0.1600277	-0.1532824	-0.1966709
**DIAGNOSTIC**	-0.0720313	-0.0122062	-0.0902056
**DIFFICULT**	0.12358507	0.10445398	0.15857958
**DIFFICULTY**	0.21643011	0.2428037	0.27675993
**DIGIT**	-0.0218749	-0.026181	0.14912271
**DIMENSION**	0.0343217	0.00616756	0.16001181

**DIMENSIONAL**	-0.1384726	-0.1222131	0.08218525
**DIRECTIONS**	-0.1239203	-0.1425482	0.00915008
**DISABILITY**	-0.0752917	-0.0496583	-0.0476038
**DISCRIMINATE**	0	0.03377596	0.06627838
**DISCRIMINATED**	0	-0.0054075	0.07310848
**DISCRIMINATION**	0.0602192	0.07415429	0.19944415
**DISCRIMINATION TASK**	0.01710306	0.0557775	0.17018883
**DISCRIMINATIVE**	-0.0370131	0.0587726	-0.0088254
**DISEASE**	-0.244464	-0.1797924	-0.2183536
**DISEASE AD**	-0.1701856	-0.1685872	-0.1577298
**DISEASE PD**	-0.1008174	-0.0439653	-0.0551422
**DISEASES**	-0.1462789	-0.1276731	-0.1658865
**DISGUST**	-0.0885813	-0.0586413	-0.0929529
**DISORDER**	-0.1574422	-0.1185065	-0.3237945
**DISORDER ADHD**	-0.0449922	-0.025453	-0.1021664
**DISORDER MDD**	-0.1233387	-0.13196	-0.2123279
**DISORDER PTSD**	-0.1070695	-0.0811526	-0.1507209
**DISORDERS**	-0.1707055	-0.0935177	-0.2843871
**DISTRACTION**	0.08651929	0.11238643	0.08356456
**DISTRACTOR**	0.10232546	0.08444067	0.17283518
**DISTRACTORS**	0.07414125	0.1009397	0.14951485
**DISTRESS**	0.01888561	0.01554401	-0.0840051
**DISTURBANCES**	-0.075445	-0.0482555	-0.1087679
**DMN**	-0.1915341	-0.1510953	-0.1891472
**DOMAIN GENERAL**	0.23920387	0.18671167	0.11858188
**DOPAMINE**	-0.0861674	0.06151927	-0.0647754
**DOPAMINERGIC**	-0.0974588	0.00811271	-0.089477
**DORSAL ATTENTION**	-0.0229069	0.00243957	0.11416326
**DRIVE**	-0.0433747	-0.0550927	-0.1202993
**DRUG**	-0.1775466	-0.0607795	-0.1772547
**DRUGS**	-0.0471404	0	-0.0348848
**DURATION**	-0.1018461	-0.017504	-0.0930546
**DYNAMIC**	0	-0.0691245	0.03380409
**DYSLEXIA**	0.1100745	0	0.01736504
**DYSREGULATION**	-0.0752104	-0.0596446	-0.1549631
**EARLY STAGE**	-0.1050982	-0.0403073	0
**EARLY STAGES**	0.05832456	0.07348196	0.13345288
**EARLY VISUAL**	-0.0846291	-0.1349214	0.09172133
**EASY**	0.05571269	0.05108777	0.06325566
**EATING**	-0.0833079	-0.0825763	-0.1293799
**ECONOMIC**	-0.0221305	0.0425154	-0.0801453
**EDUCATION**	-0.0469059	-0.0894558	-0.0505769
**EFFICIENT**	0.0865907	0.07401774	0.20966366
**EFFORT**	0.08022563	0.12495086	0.12336133

**EFFORTFUL**	0.10488991	0.11880123	0.08061789
**ELDERLY**	-0.0396395	-0.0657277	-0.0066296
**EMOTION**	-0.0360986	-0.0790027	-0.2775634
**EMOTION REGULATION**	0	0	-0.1852976
**EMOTIONAL**	-0.0253391	-0.0739478	-0.271591
**EMOTIONAL FACES**	0	-0.0426297	-0.0777579
**EMOTIONAL INFORMATION**	0.05544382	0.03583049	-0.0380138
**EMOTIONAL RESPONSES**	0	0	-0.1555655
**EMOTIONAL STIMULI**	-0.0724447	-0.0835408	-0.1659416
**EMOTIONAL VALENCE**	-0.0424002	-0.102059	-0.1400235
**EMOTIONALLY**	-0.0539848	-0.1160902	-0.1726948
**EMOTIONS**	-0.0169976	-0.1030267	-0.241628
**EMPATHIC**	0.08381712	0.06602294	-0.0672494
**EMPATHY**	0.14852857	0.10300627	-0.0219668
**ENCODE**	-0.0561405	-0.078056	0.05608502
**ENCODED**	0.02599772	-0.0124416	0.08270153
**ENCODING**	0.06779344	0	0.10064031
**ENCODING RETRIEVAL**	0.04042552	0.03858268	0.0662881
**ENVIRONMENT**	-0.0218077	-0.0075788	0.15836736
**ENVIRONMENTAL**	0	0.01727009	-0.027783
**ENVIRONMENTS**	0.02266933	0.01792324	0.05952151
**EPILEPSY**	-0.0817986	-0.113631	-0.1935799
**EPISODIC**	-0.0072842	-0.073656	-0.0975206
**EPISODIC MEMORY**	-0.0171393	-0.0828293	-0.0871482
**ERROR**	0.09964015	**0.30530341**	0.03370881
**ERRORS**	0.07182664	0.25124055	0.03502916
**ESTIMATE**	-0.0759255	-0.0510241	-0.052267
**ESTIMATED**	-0.1230595	-0.1027991	-0.1034222
**ESTIMATES**	-0.0687196	-0.0665955	-0.0759831
**ESTIMATION**	0	0.09650864	0.06001513
**EVALUATE**	-0.0486602	-0.0239419	-0.0501851
**EVALUATED**	-0.0383433	-0.0418906	-0.0890357
**EVALUATING**	-0.020484	-0.0017657	-0.0911436
**EVALUATION**	-0.0200142	-0.007937	-0.1684219
**EVALUATIONS**	-0.0038188	-0.0604886	-0.1312986
**EXACT**	0.0401171	0.0536917	0.1184332
**EXECUTED**	-0.035984	-0.0409085	0.08546923
**EXECUTION**	0.03232047	0.07832307	0.25386671
**EXECUTIVE**	0.14399767	0.26872788	0.14810277
**EXECUTIVE CONTROL**	0.07215874	0.11881911	0.10520474
**EXECUTIVE FUNCTION**	0.08380132	0.09145224	0.0561605
**EXECUTIVE FUNCTIONS**	0.09016697	0.2143544	0.0849544
**EXERT**	-0.0077755	0	0.03839403
**EXPECTANCY**	0.09090806	0.18253627	0.08852836

**EXPECTATION**	-0.0020232	0.05705257	-0.0111478
**EXPECTATIONS**	-0.0129624	-0.017774	-0.0632561
**EXPECTED**	-0.0440636	0.01538143	0.00105441
**EXPERIENCE**	-0.0310865	0	-0.059593
**EXPERIENCED**	-0.0571192	0.01463195	-0.1455978
**EXPERIENCES**	-0.0561041	-0.040005	-0.0994163
**EXPERIENCING**	-0.0042214	0.0332549	-0.080777
**EXPERTISE**	0.00567946	-0.0414494	0.11921758
**EXPLICIT**	0.10041343	0	0.00599769
**EXPLICITLY**	0.04714682	0.03416996	-0.0404929
**EXPLORATION**	-0.0464618	-0.0529501	0.0505377
**EXPLORE**	-0.022919	-0.0473862	-0.111164
**EXPOSED**	-0.0153677	-0.0467057	-0.1258087
**EXPOSURE**	-0.0899831	-0.1121268	-0.1255114
**EXPRESSED**	0.03949157	-0.0073223	-0.0507611
**EXPRESSION**	-0.0735406	-0.0812873	-0.1106677
**EXPRESSIONS**	0.01461698	-0.1103085	-0.1565304
**EXTERNAL**	-0.0734158	0	-0.0364082
**EXTERNALLY**	0	0.09540978	0.06530921
**EXTINCTION**	-0.0735208	0.02730793	-0.1173857
**EYE**	-0.0318503	-0.004474	0.26878487
**EYE MOVEMENT**	-0.0820954	-0.0576804	0.06021333
**EYE MOVEMENTS**	-0.0531466	-0.0640428	0.21115564
**EYES**	-0.1266437	-0.1485225	0.00494477
**FACE**	0.004132	-0.1282904	0.00575171
**FACE RECOGNITION**	-0.0598311	-0.1290107	0.01541987
**FACES**	-0.0460058	-0.1593065	-0.0594788
**FACIAL**	-0.0216397	-0.1419148	-0.1841119
**FACIAL EXPRESSION**	-0.0535145	-0.100852	-0.0680893
**FACIAL EXPRESSIONS**	-0.0004165	-0.1170941	-0.1567209
**FACT**	0.00378715	0	0.00876717
**FAILED**	0.04693833	0.11463222	0.11256292
**FAMILIAR**	0.0747552	-0.0588338	0.17204498
**FAMILIARITY**	0.04832977	-0.0308282	0.1133847
**FEAR**	-0.1041026	-0.0329673	-0.212524
**FEARFUL**	-0.0646407	-0.1086965	-0.1316286
**FEARFUL FACES**	-0.0843718	-0.1027587	-0.0954597
**FEEDBACK**	-0.0099334	0.12683825	0
**FEELING**	0.01208524	0.01093414	-0.0719485
**FEELINGS**	-0.0233269	-0.0375989	-0.1514856
**FEMALE**	-0.1342963	-0.0932882	-0.1869324
**FEMALES**	-0.0854186	0	-0.1046874
**FINGER**	-0.0841008	-0.0318346	0.07700133
**FINGER MOVEMENTS**	-0.046366	-0.0087419	0.12009711

**FINGER TAPPING**	-0.0538838	0	0.04668296
**FIXATION**	-0.067992	-0.1058879	0.13509146
**FIXED**	-0.0501949	-0.0190655	-0.030967
**FLEXIBILITY**	0.04116971	0.09046347	0.10453099
**FLEXIBLE**	0.11895268	0.16911064	0.20353777
**FLEXIBLY**	0.06326359	0.10430362	0.16109594
**FLOW**	-0.2232631	-0.1712972	-0.1290333
**FLUENCY**	0.16595854	0.13161923	0
**FOCUS**	0.00066145	0.01389567	0.00335178
**FOCUSED**	0	0.06558626	-0.0470405
**FOCUSING**	0.0175886	0.07349826	-0.0088628
**FOOD**	-0.1142302	-0.0420319	-0.1004065
**FOOT**	-0.0672033	0	-0.0297408
**FORCE**	-0.0548792	-0.0146182	0.01264845
**FORM**	0.11947111	-0.0462296	0.1190783
**FUNCTIONAL ABNORMALITIES**	-0.0382353	-0.0589046	-0.1200925
**FUTURE**	-0.1349725	-0.0739289	-0.1416068
**GAIN**	-0.0586606	0.06086013	-0.0507905
**GAINS**	-0.0575532	0.04686272	-0.0877839
**GAMBLING**	-0.0520401	0.03068269	-0.0954305
**GAME**	0	0.08990519	-0.0410733
**GAZE**	-0.0525004	-0.1301284	0.09180636
**GENDER**	-0.0485295	-0.0814872	-0.2307124
**GERMAN**	0.19087374	0.10743882	-0.0177803
**GESTURES**	0.10562154	-0.0480918	0.15892322
**GOAL**	0	0.06395579	0.170948
**GOAL DIRECTED**	0.0025953	0.08303134	0.10541052
**GOALS**	-0.0082868	0.01909105	0.08143615
**GOOD**	-0.0582174	-0.065655	-0.0325366
**GRASPING**	0.00970705	-0.0189492	0.24869276
**GROUP HEALTHY**	-0.0503434	-0.0460688	-0.0983133
**HAND**	-0.0710688	-0.0542194	0.14318929
**HAND MOVEMENTS**	-0.0708957	-0.0964532	0.03717734
**HANDS**	-0.0427863	-0.0670819	0.15641359
**HAPPY**	-0.0882539	-0.147363	-0.2054107
**HAPPY FACES**	-0.1124527	-0.1397583	-0.1633668
**HEALTHY**	-0.2160435	-0.0633425	-0.2752597
**HEALTHY ADULTS**	0.02447622	0.07274782	0.02046273
**HEALTHY CONTROL**	-0.0800618	-0.0152947	-0.0888366
**HEALTHY CONTROLS**	-0.2052108	-0.1599157	-0.2949584
**HEALTHY HUMAN**	-0.0500473	0.00765291	-0.050169
**HEALTHY INDIVIDUALS**	-0.1014469	-0.0570612	-0.1262598
**HEALTHY MALE**	-0.001993	0.01213658	0.00178705
**HEALTHY VOLUNTEERS**	-0.0247271	0.09763792	0

**HEALTHY YOUNG**	-0.0286539	-0.0096414	-0.0158349
**HEARD**	0.11048045	0.03022658	-0.0990535
**HEARING**	0.09833972	0	-0.0005069
**HIGH FUNCTIONING**	0.05055854	0.03855504	0.02879178
**HIGH RISK**	-0.0448031	-0.0458028	-0.1562513
**HYPERACTIVATION**	0.07268258	0.06542293	0.06109298
**HYPERACTIVITY**	-0.1020688	-0.0430537	-0.1303702
**HYPERACTIVITY DISORDER**	-0.0697944	-0.0276706	-0.1177681
**IDENTITY**	0.05713186	-0.0121675	0.08666438
**ILLNESS**	-0.1233455	-0.1101813	-0.2277133
**ILLUSION**	-0.0431779	-0.0550806	0.10187988
**IMAGE**	-0.166703	-0.2229555	-0.0927594
**IMAGERY**	-0.0059277	0	0.20832568
**IMAGES**	-0.1744124	-0.2050329	-0.0837185
**IMAGINE**	0	0.00843549	0
**IMAGINED**	-0.0448472	-0.0385337	0.0703664
**IMITATION**	0.05556201	0.02511029	0.15439503
**IMPAIRED**	-0.0451255	-0.0631885	-0.1117562
**IMPAIRMENT**	-0.0652299	-0.0774032	-0.1040148
**IMPAIRMENTS**	0.00930783	-0.0039304	-0.0471343
**IMPLICIT**	-0.0526731	-0.1110015	-0.0879688
**IMPULSIVE**	-0.0268803	0.00989394	-0.0585321
**IMPULSIVITY**	-0.0605321	0.02759891	-0.1040665
**INCENTIVE**	-0.0660719	0.08551237	-0.0655521
**INCENTIVE DELAY**	-0.0846115	0.05642253	-0.114708
**INCONGRUENT**	0.19958325	0.14997735	0.15773263
**INCONSISTENT**	0.01234433	-0.0070611	-0.0425269
**INCORRECT**	0.01575236	0.0395046	-0.0019885
**INDEX FINGER**	-0.0926846	-0.0374912	0.02086551
**INDUCTION**	-0.0074102	0	-0.0282762
**INFERENCE**	0.01177262	-0.0613801	-0.0506223
**INFERENCES**	0.07632149	0	-0.0764576
**INFORMATION**	0.18898222	0.0959739	0.26290743
**INHIBIT**	0.10506955	0.19624303	0.07723733
**INHIBITION**	0.11399799	0.28038679	0.01742586
**INHIBITORY**	0.0382911	0.18307948	-0.0173065
**INHIBITORY CONTROL**	0.09521373	0.17184433	0.04583307
**INITIATION**	-0.0872076	-0.0431113	-0.0237653
**INJURY**	0	0.01484875	-0.0040591
**INTEGRATE**	0.06554485	0.08268082	0.11951937
**INTEGRATION**	0.02736221	-0.0555899	0
**INTEGRATIVE**	0.07936534	0.02393315	0.04240474
**INTEGRITY**	-0.1570609	-0.183176	-0.2194648
**INTELLIGENCE**	-0.0325796	-0.0660114	-0.0282174

**INTENSE**	-0.0085017	0.10892197	-0.0385577
**INTENSITY**	-0.10914	0.03710656	-0.1636815
**INTENTION**	0.00638838	-0.0559815	-0.0450303
**INTENTIONAL**	0.04198949	0.02371044	-0.0342385
**INTENTIONS**	0.07979495	-0.0260683	-0.0332344
**INTERACTIVE**	0	-0.0393941	-0.0342861
**INTERFERENCE**	0.20823567	0.25831132	**0.31039411**
**INTERNAL**	-0.0055759	0	0.04558844
**INTERNALLY**	0.02604751	0.06217858	0.09179886
**INTEROCEPTIVE**	-0.0442138	0.02011439	-0.1055506
**INTERPERSONAL**	0.02598102	0.04818906	-0.0783581
**INTERPRET**	0	-0.0114211	0
**INTERVAL**	0	0.02653552	0.04727741
**INTERVALS**	-0.018162	0.02249871	0
**INTERVENTION**	-0.0588753	-0.0642993	-0.1018453
**INTERVENTIONS**	-0.1118863	-0.1058652	-0.1547168
**INTRINSIC**	-0.1384743	-0.1024518	-0.1574449
**INTRINSIC FUNCTIONAL**	-0.067918	-0.0176146	-0.0797945
**INVOLVED COGNITIVE**	0.11537109	0.13988922	0.02171795
**IQ**	-0.0259712	-0.078688	-0.0979809
**IRRELEVANT**	0.15079966	0.12350857	**0.30862378**
**JUDGE**	0.12488197	0.04975057	0.02472505
**JUDGED**	0.05314231	0.01843555	0.05981918
**JUDGMENT**	0.25564194	0.08576416	0.1531998
**JUDGMENT TASK**	0.19062197	0.0475112	0.08760708
**JUDGMENTS**	0.14560933	0.06619682	0.05166935
**KNOWLEDGE**	0.16253007	-0.0209594	0.01334066
**LANGUAGE**	**0.35602538**	0.08487029	0.02697316
**LANGUAGE COMPREHENSION**	0.20392043	-0.006922	-0.0820014
**LANGUAGE NETWORK**	0.22264418	0.06012153	0
**LANGUAGES**	0.29023471	0.10303417	0.10434025
**LATENCIES**	-0.0157533	0	0.04429326
**LATENCY**	-0.0571254	-0.0313644	0.0111112
**LEARN**	0	0.02406861	0
**LEARNED**	-0.0076723	0.03859097	0.064984
**LEARNING**	-0.0721867	0.01618679	0
**LEARNING TASK**	-0.0513037	0.07399599	-0.0643853
**LESION**	0	-0.0929637	0.02188614
**LESIONS**	-0.0743717	-0.1231792	-0.0368576
**LETTER**	0.15782047	0.08692318	0.24285463
**LETTERS**	0.07689756	0.01507659	0.26705427
**LEXICAL**	**0.31418528**	0.09900944	0.06490569
**LEXICAL DECISION**	0.15896895	0.02824969	0.07147725
**LIMB**	-0.1242932	-0.10752	0

**LINGUISTIC**	**0.32255864**	0.09335934	0.05495849
**LISTENED**	0.09329929	0	-0.1004709
**LISTENING**	0.11239451	0	-0.1059012
**LOAD**	0.18729027	0.26116602	**0.35448846**
**LONG TERM**	0.00712512	-0.0109665	0.0010166
**LOSS**	-0.205161	-0.1224389	-0.2719119
**LOSSES**	-0.0399852	0.11467072	-0.0688493
**MAINTAIN**	0.11161686	0.15257214	0.12133417
**MAINTAINED**	0.05529929	0.09115929	0.20475725
**MAINTAINING**	0.04164485	0.0909181	0.14322547
**MAINTENANCE**	0.18569581	0.25290523	0.28123579
**MAJOR DEPRESSION**	-0.0396714	-0.0461102	-0.1265957
**MAJOR DEPRESSIVE**	-0.1277432	-0.1438501	-0.2294729
**MALE**	-0.0929532	-0.0892378	-0.1566988
**MALE FEMALE**	-0.0856266	-0.1112142	-0.1228597
**MALES**	-0.0679992	-0.0294301	-0.126926
**MANIPULATED**	0.18207114	0.12725068	0.17484139
**MANIPULATING**	0.15311649	0.08444776	0.11364685
**MANIPULATION**	0.15194445	0.14114515	0.26278607
**MANIPULATIONS**	0.08779394	0.08404572	0.09513099
**MATCH**	0.02245583	0.04538231	0.08330615
**MATCHING**	0.13953745	0.06919184	0.20971162
**MATCHING TASK**	0.12816653	0.04980094	0.15000786
**MATURATION**	-0.0007113	0.01966033	0
**MCI**	-0.0981997	-0.1411045	-0.0928447
**MDD**	-0.1279893	-0.1310354	-0.2202411
**MEANING**	0.27119995	0.05641447	0
**MEDICATION**	-0.1036352	-0.0616558	-0.1780803
**MEMORIES**	-0.0471455	-0.1281552	-0.1380964
**MEMORY**	0.15183704	0.14056483	0.20485993
**MEMORY ENCODING**	0.04473225	-0.0074506	0.05466459
**MEMORY LOAD**	0.17956233	0.21391756	0.29398107
**MEMORY PERFORMANCE**	0.01803301	0.00704674	0.0147349
**MEMORY PROCESSES**	0.07893713	0.09774107	0.09440235
**MEMORY RETRIEVAL**	0.06788239	0.08273798	-0.0077106
**MEMORY TASK**	0.1129197	0.17557144	0.16165939
**MEMORY TASKS**	0.17519266	0.1808663	0.11637017
**MEMORY WM**	0.16954693	0.25003122	**0.33880899**
**MEN**	-0.0531117	0.0224423	-0.1518796
**MEN WOMEN**	-0.0381507	0.03148968	-0.1162854
**MENTAL**	0.03435533	-0.0188228	0.14978688
**MENTAL IMAGERY**	0.01223625	0	0.16775065
**MENTAL STATE**	0	-0.0660161	-0.1224808
**MENTAL STATES**	0.02415969	-0.0972248	-0.1434271

**MENTALIZING**	0.04461854	-0.0749056	-0.0487495
**METABOLISM**	-0.1579277	-0.1184888	-0.1614277
**MILD COGNITIVE**	-0.0834277	-0.097953	-0.0905352
**MIND**	0.05993991	-0.0488128	-0.131716
**MIND TOM**	0.06875448	-0.0567398	-0.118744
**MIRROR**	0.02786392	-0.0736686	0.16974094
**MIRROR NEURON**	0.04167964	-0.0523681	0.13905212
**MNEMONIC**	0.0901332	0.08381736	0.06944649
**MODULATE**	-0.0153108	0.01894572	-0.032374
**MODULATES**	0.03078905	0.06177224	0
**MODULATING**	-0.0383025	0.02108653	-0.0799814
**MODULATION**	-0.0961425	-0.0121884	-0.0998052
**MODULATIONS**	-0.036192	-0.0277829	0.00650394
**MODULATORY**	-0.0702169	-0.0048065	-0.074975
**MONETARY**	-0.1043714	0.07073199	-0.1387874
**MONETARY REWARD**	-0.0744214	0.06882484	-0.0616677
**MONEY**	-0.1075038	0.00676713	-0.0800365
**MONITOR**	0.10286043	0.15248923	0.0540049
**MONITORED**	-0.0404198	-0.0164171	0.03075499
**MONITORING**	0.13091657	0.28217545	0.07975072
**MOOD**	-0.0831398	-0.1161301	-0.218198
**MORAL**	-0.0003049	-0.0451729	-0.1469012
**MOTION**	-0.0681057	-0.1632354	0.14585047
**MOTIVATION**	-0.0504664	0.07199418	-0.0938103
**MOTIVATIONAL**	-0.0284611	0.09062176	-0.0863425
**MOTOR**	-0.0431152	0.01793294	0.10714981
**MOTOR CONTROL**	-0.0542364	-0.0136223	0.05847926
**MOTOR IMAGERY**	-0.0193437	0.01338353	0.16080257
**MOTOR NETWORK**	-0.0908434	0	0
**MOTOR PERFORMANCE**	-0.1015461	-0.0649157	0.00473106
**MOTOR RESPONSE**	0.05621343	0.12624044	0.09435459
**MOTOR RESPONSES**	0	0.07501166	0.04955593
**MOTOR TASK**	-0.0595976	-0.0243289	0.03609848
**MOVEMENT**	-0.1191347	-0.0806765	0.03641125
**MOVEMENTS**	-0.0882105	-0.0855701	0.14196545
**MOVING**	-0.0416729	-0.0890148	0.20091709
**MULTIPLE SCLEROSIS**	0	0.03139478	0.09687653
**MULTISENSORY**	-0.0234369	-0.0646149	0.10657725
**MUSCLE**	-0.1276201	-0.0491885	-0.0873189
**MUSIC**	0.04518853	0	-0.0649813
**MUSICAL**	0.04736714	0.01856207	-0.0551006
**MUSICIANS**	0.07982409	0.06436737	0.06285245
**NAMES**	0.03167522	-0.0502015	-0.028646
**NAMING**	0.11790483	0	0.04442348

**NATURAL**	0.00934555	-0.1172243	-0.0128344
**NATURALISTIC**	-0.034929	-0.1170189	-0.0535186
**NATURE**	0.03987276	0	0.0943385
**NAVIGATION**	-0.0631501	-0.038765	0.11372459
**NEGATIVE**	-0.1138206	-0.0955647	-0.2790509
**NEGATIVE AFFECT**	-0.003784	0.01340322	-0.1406546
**NEGATIVE EMOTIONAL**	-0.0700852	-0.0718076	-0.131527
**NEGATIVE NEUTRAL**	0.02842521	-0.0423805	-0.0803404
**NEGATIVE POSITIVE**	-0.0418422	-0.035874	-0.1291149
**NEGATIVITY**	-0.0449288	0	-0.0664779
**NERVOUS**	-0.1385404	-0.0453048	-0.0567742
**NETWORK DMN**	-0.1928997	-0.1570978	-0.2051958
**NEURODEGENERATIVE**	-0.10585	-0.1230607	-0.1432343
**NEURODEVELOPMENTAL**	-0.0747278	-0.080122	-0.0863096
**NEUROPSYCHIATRIC**	-0.0552453	-0.0502894	-0.1216976
**NEUROPSYCHOLOGICAL**	0	-0.0250384	0.05315754
**NEUTRAL**	-0.0435719	-0.1010577	-0.1620952
**NEUTRAL FACES**	-0.0861193	-0.1099694	-0.1017318
**NEUTRAL PICTURES**	-0.029101	-0.0611406	-0.1086116
**NEUTRAL STIMULI**	-0.0213471	0	-0.0864767
**NEW**	-0.0040209	1.27E-06	0.03564179
**NEWLY**	-0.0545135	-0.0393472	0.01116555
**NOCICEPTIVE**	-0.0089807	0.14289735	-0.0639534
**NOGO**	0.06053578	0.14061077	0.02355219
**NORMAL CONTROLS**	-0.0447188	-0.0441755	-0.0663679
**NOUNS**	0.13264031	0.02102308	-0.0552794
**NOVEL**	0.05280151	0.06005229	0.03594515
**NOVELTY**	0.03924283	0.06536884	0.0649019
**NOXIOUS**	0	0.18780969	-0.0553414
**NUMBER**	0.05382135	0.11334559	0.23184442
**NUMBERS**	0.13505927	0.14569301	**0.37867394**
**NUMERICAL**	0.09171472	0.12137934	**0.36131194**
**NUMEROUS**	0	0.00890854	-0.0265769
**OBJECT**	0.03809476	-0.1158586	**0.33054492**
**OBJECT RECOGNITION**	-0.0479153	-0.1357814	0.11653459
**OBJECTS**	0	-0.1401154	0.28851434
**OBSERVATION**	0.04904845	-0.0379106	0.20620886
**OBSERVATIONS**	0.03545427	0.00894026	0.04340155
**OBSERVING**	0.08920639	-0.0214436	0.10562364
**OBSESSIVE**	-0.0550794	-0.0073486	-0.1220226
**OBSESSIVE COMPULSIVE**	-0.0526272	-0.003433	-0.1183003
**OCD**	-0.0498532	-0.0041399	-0.0974744
**ODDBALL**	0.02753909	0.08942837	0.00675037
**OLD**	-0.0269778	-0.0081384	-0.0321692

**OLDER**	0.00632269	0.02477362	-0.027346
**OLDER ADULTS**	0.00861697	0.00953711	0
**OLFACTORY**	-0.0658807	0.00562175	-0.0965198
**OPPOSED**	-0.0355894	-0.0198198	-0.0362332
**OPPOSITE**	-0.1142609	-0.0543983	-0.0180675
**ORAL**	-0.0029143	-0.0483623	-0.039357
**ORIENTATION**	-0.1141462	-0.1022427	0.13329482
**ORIENTED**	-0.0176882	-0.0056842	0.11195822
**ORIENTING**	0.08022773	0.10297655	0.26597521
**ORTHOGRAPHIC**	0.24616868	0.09594863	0.18538813
**OSCILLATIONS**	-0.1602225	-0.1324223	-0.1232289
**OUTCOME**	-0.0214341	0.09004339	-0.1437062
**OUTCOMES**	-0.0947293	0.00961911	-0.1655917
**OUTPUT**	-0.0441208	-0.0158923	0
**OVERT**	0.08839886	0.0773594	0.10337469
**PACED**	0	0.01670546	0.09544858
**PAIN**	-0.0590787	0.18377373	-0.081629
**PAINFUL**	0	0.2413732	-0.0017003
**PAIR**	0.05371351	0	0.02279995
**PAIRED**	-0.047445	-0.0205098	-0.063407
**PAIRS**	0.17997908	0.07749963	0.09262237
**PARADIGM**	0.08964118	0.16253271	0.11934199
**PARADIGMS**	0.08247138	0.11600246	0.05454843
**PARKINSON**	-0.1218267	-0.0521439	-0.0609616
**PARKINSON DISEASE**	-0.1179623	-0.0502749	-0.0628842
**PARTNER**	0	0.07646877	-0.0533938
**PASSIVE**	-0.025038	-0.0603703	-0.0469827
**PASSIVE VIEWING**	-0.0306721	-0.0449149	0.0318782
**PASSIVELY**	0.02755715	-0.0740451	0.05459409
**PAST**	-0.0712799	-0.1057398	-0.1305755
**PATHOLOGICAL**	-0.1360961	-0.1012094	-0.183665
**PATHOLOGY**	-0.1262653	-0.107449	-0.1818317
**PATHOPHYSIOLOGICAL**	-0.0802103	-0.0783388	-0.1449319
**PATHOPHYSIOLOGY**	-0.1575347	-0.115718	-0.2215931
**PATIENT**	-0.1274045	-0.162529	-0.1611182
**PATIENT GROUP**	-0.0145849	-0.0004864	-0.0526556
**PATTERN**	0.02923606	0.03563509	0.14238403
**PATTERNS**	0	0.0144297	0.07595335
**PD**	-0.1260294	-0.0725865	-0.0885682
**PERCEIVE**	0	-0.0234241	-0.0253935
**PERCEIVED**	-0.0065554	0.02446525	0
**PERCEIVING**	0	-0.0707475	0.01863194
**PERCEPT**	0	0	0.12507214
**PERCEPTION**	0	-0.0870391	0.07062581

**PERCEPTUAL**	0.06760754	-0.0219706	0.26825863
**PERFORM**	0.07441812	0.11565931	0.22791372
**PERFORMANCE**	0.10929956	0.18490865	0.26679643
**PERFORMANCE TASK**	0.05470908	0.09118276	0.09751118
**PERFORMANCES**	0.02321462	-0.0037779	-0.0243039
**PERFORMING**	0.06968265	0.08276168	0.17893219
**PERSISTENT**	0	0.00467551	-0.0810446
**PERSONAL**	-0.0341504	-0.0696214	-0.1511705
**PERSONALITY**	-0.0374588	-0.0583061	-0.1777325
**PERSONALITY TRAITS**	-0.0616144	-0.089461	-0.1855561
**PERSPECTIVE**	0.04496785	0.010021	0.03637643
**PHARMACOLOGICAL**	-0.0337329	0.07207268	-0.0550876
**PHONOLOGICAL**	**0.36165551**	0.17216819	0.17783058
**PHOTOGRAPHS**	-0.0874798	-0.0878439	-0.0497882
**PHYSICAL**	-0.0352531	-0.0602695	0.04853005
**PICTURE**	0.0362965	0	0
**PICTURES**	-0.011402	-0.0275844	-0.0709113
**PITCH**	0.08226733	0.06583694	0
**PLACE**	-0.0199269	-0.0670398	0
**PLACEBO**	-0.1268152	0.00164775	-0.1548991
**PLACEBO CONTROLLED**	-0.0784515	0	-0.0679181
**PLANNING**	0.04128516	0.08222239	0.23291478
**PLASTICITY**	-0.0547966	-0.1207152	-0.0247285
**PLEASANT**	-0.0165862	-0.0034841	-0.1622748
**POINT**	-0.0867254	-0.076698	-0.0115364
**POINTING**	0.04203557	0.05431413	0.15643983
**POINTS**	-0.109314	-0.0634539	-0.0719216
**POLYMORPHISM**	-0.0828248	-0.0395679	-0.1300769
**POSITION**	-0.0193786	-0.0560132	0.16956533
**POSITIVE**	-0.1495337	-0.1384016	-0.3066775
**POSTTRAUMATIC**	-0.1118052	-0.101253	-0.1419014
**PRACTICE**	-0.0863586	-0.0776453	0.01734776
**PREDICT**	-0.0596987	-0.0573038	-0.0237422
**PREDICTED**	-0.0540568	-0.0177415	-0.1276138
**PREDICTING**	-0.0268115	0	-0.0700281
**PREDICTION**	0.03619407	0.06178179	0.02567493
**PREDICTION ERROR**	0.08333796	0.16945685	0.0075745
**PREDICTIONS**	0.11797376	0.08891468	0.10851659
**PREDICTIVE**	-0.0377829	-0.0281576	-0.0299932
**PREDICTS**	0.02279169	0	-0.0242287
**PREFERENCE**	-0.0385347	-0.0280724	0.02912356
**PREFERENCES**	-0.0014423	0.04188474	-0.0661298
**PREFERENTIAL**	0.04795691	0.01524863	0.07761338
**PREFERENTIALLY**	0.02968272	-0.0268989	0.09152949

**PREPARATION**	0	0.10690469	0.19571314
**PREPARATORY**	0	0.1076736	0.21444821
**PRESS**	0.04771314	0.12767611	0.09401625
**PRESSURE**	-0.0306435	0.0676919	-0.088612
**PRIME**	-0.0471476	-0.0686656	0.01665361
**PRIMING**	0.07457785	-0.060736	0
**PROBLEM**	-0.004119	0.04363964	0
**PROBLEMS**	0.01039334	0.04750308	-0.0233279
**PROSPECTIVE**	-0.0423054	0.03120214	-0.0407586
**PSEUDOWORDS**	0.17750603	0.03705773	0.10268172
**PSYCHIATRIC**	-0.1076241	-0.0328625	-0.1897343
**PSYCHIATRIC DISORDERS**	-0.0539228	0.01950567	-0.1173457
**PSYCHOPATHOLOGY**	-0.0861278	-0.0182836	-0.1618169
**PSYCHOSIS**	-0.039439	-0.0240197	-0.1695913
**PSYCHOTIC**	-0.076668	-0.0472535	-0.1237253
**PTSD**	-0.1187998	-0.0937326	-0.1678187
**PUNISHMENT**	0	0.09703271	-0.1088371
**RAPID**	-0.0244541	0.04737981	0.0722294
**RATE**	-0.149599	-0.0511159	-0.099334
**RATED**	-0.0255406	0.01189113	-0.1145793
**RATES**	-0.0283335	0.0360571	-0.0622284
**RATING**	-0.1015207	-0.0762081	-0.2237743
**RATINGS**	-0.052627	0.05763277	-0.206995
**REACH**	-0.1044154	-0.06385	0.04881421
**REACHING**	-0.0691617	-0.0382793	0.11302032
**REACTION**	0.04791797	0.16041056	0.20310212
**REACTION TIME**	0.04406068	0.14673785	0.18787661
**REACTION TIMES**	0.06641187	0.11334766	0.15151539
**REACTIONS**	-0.0628864	0	-0.129854
**REACTIVITY**	-0.1270684	-0.0926295	-0.2108303
**READ**	0.18517889	0	0.03142728
**READERS**	0.23184319	0.0761256	0.14506816
**READING**	0.260283	0.03826123	0.17116926
**REAPPRAISAL**	0.09794499	0.06655125	-0.0835833
**REASONING**	0.08565322	0.06476557	0.05976827
**RECALL**	0.05324885	0.01801877	-0.0646858
**RECOGNITION**	0.11131362	-0.0288058	0.09761692
**RECOGNITION MEMORY**	0.05451391	0.05330158	0.03424743
**RECOGNITION TASK**	0.02232012	0.04764454	0.0371778
**RECOGNIZE**	0	-0.0992266	0.00696522
**RECOGNIZED**	0.00604695	0	0
**RECOGNIZING**	0.0864008	0	-0.0178967
**RECOLLECTION**	-0.0388375	-0.0376418	-0.0625391
**RECORDING**	-0.0893901	0.00386895	-0.0786689

**RECORDINGS**	-0.135066	-0.1154039	-0.0871784
**REFERENTIAL**	-0.0505087	-0.1039134	-0.169094
**REGULATE**	0	0	-0.1444549
**REGULATING**	0	0.04500807	-0.1198594
**REGULATION**	-0.0557279	0	-0.2679717
**REGULATORY**	0.048506	0.10788312	-0.0726184
**REHABILITATION**	-0.0709561	-0.0756479	-0.035676
**REHEARSAL**	0.12260677	0.11801692	0.16727399
**REINFORCEMENT**	-0.0593988	0.02221566	-0.0995634
**REMEMBER**	0.01356887	0.00181596	0.06193361
**REMEMBERED**	-0.0322752	-0.1033731	-0.0343435
**REMEMBERING**	0	-0.021169	-0.0229725
**REPEAT**	-0.0537258	-0.0295768	-0.0005055
**REPEATED**	-0.0070056	-0.0517229	0.08769929
**REPETITION**	0.10464105	-0.0372358	0.07584297
**REPETITION SUPPRESSION**	0.09458356	-0.0492792	0.16714416
**REPETITIVE**	-0.0251231	-0.0420555	-0.0130184
**REPRESENTATION**	0.01458315	-0.0946416	0.21365907
**REPRESENTATIONAL**	0.00818323	-0.078891	0.06099766
**REPRESENTATIONS**	0.17001789	0	0.28825002
**RESOLVE**	0.08905513	0.09625575	0.03953718
**RESPOND**	0.04832357	0.06272697	0.08185515
**RESPONDED**	0.08735605	0.05521327	0.08985758
**RESPONDING**	0.04714395	0.10569378	0
**RESPONDS**	0.0346103	-0.0624212	0.01869647
**RESPONSE**	0	0.17324345	0
**RESPONSE INHIBITION**	0.18069767	**0.32170642**	0.08887237
**RESPONSE SELECTION**	0.10731938	0.20202506	0.2031505
**RESPONSE TIME**	0.12546718	0.14209453	0.18866987
**RESPONSE TIMES**	0.12362841	0.15283584	0.2151043
**RESPONSES**	-0.0484129	0.08246007	-0.1354936
**RESPONSIVE**	-0.0289695	-0.0931919	0.03105811
**RESPONSIVENESS**	-0.0734061	-0.0237969	-0.0906595
**REST**	-0.1654373	-0.1124317	-0.0964025
**RESTING**	-0.3031966	-0.2600099	-0.3407049
**RESTING STATE**	-0.2959226	-0.2557837	-0.3369009
**RETENTION**	0	0.03651001	0.07683013
**RETRIEVAL**	0.1676199	0.10573112	0.08211092
**RETRIEVED**	0.05579062	0	-0.0190786
**REVERSAL**	0.01204752	0.1100805	0
**REVERSE**	-0.0410389	0	0.09125866
**REWARD**	-0.117156	0.05183218	-0.1785189
**REWARD ANTICIPATION**	-0.1078534	0	-0.0999999
**REWARDING**	-0.0725814	-0.0063997	-0.1287767

**REWARDS**	-0.0531261	0.09175449	-0.0877545
**RHYTHM**	-0.0182099	-0.0163196	-0.006149
**RISK**	-0.1311179	-0.0274847	-0.2522215
**RISKY**	-0.0062265	0.08555145	-0.0511406
**ROTATION**	-0.0015827	-0.0128222	0.2924218
**RSFC**	-0.1305124	-0.1660165	-0.1617747
**RT**	0.08428067	0.16937106	0.20900531
**RULE**	0.15184374	0.19311423	0.20965277
**RULES**	0.17450891	0.20437717	0.20217706
**SACCADE**	-0.046969	0	0.2268608
**SACCADES**	-0.0327697	-0.0132524	0.18485827
**SAD**	-0.0803799	-0.1007213	-0.2105971
**SALIENCE**	-0.0518273	0.08822358	-0.1152096
**SALIENCE NETWORK**	-0.0283089	0.14534278	-0.0724776
**SALIENT**	0.03205502	0.06287351	0.01552535
**SCENE**	-0.1016987	-0.1674757	0
**SCENES**	-0.0635617	-0.139231	0
**SCHIZOPHRENIA**	-0.0467526	-0.0551291	-0.1991758
**SCHIZOPHRENIC**	-0.0364286	-0.0371783	-0.0978541
**SCLEROSIS**	-0.0075521	0	0.02960515
**SEARCH**	0.00788661	-0.0257534	0.08057762
**SEEKING**	-0.0669491	0.03087047	-0.0762796
**SEGREGATION**	0.0263951	0.03059368	0.13092875
**SELECT**	0.0291124	0.02917633	0.05182776
**SELECTED**	-0.0460289	-0.033309	-0.025127
**SELECTION**	0.15481877	0.20969704	0.20482476
**SELECTIVE**	0.00751099	-0.124697	0.19068102
**SELECTIVE ATTENTION**	0.04338913	0.04471188	0.25798973
**SELECTIVITY**	0	-0.1467799	0.11308963
**SELF**	-0.1025614	-0.035255	-0.1914748
**SELF REFERENTIAL**	-0.0529094	-0.101559	-0.1653597
**SEMANTIC**	**0.30165369**	0.03765843	-0.0015535
**SEMANTIC MEMORY**	0.03051079	-0.021557	-0.0713991
**SEMANTICALLY**	0.23173776	0.0256456	0
**SEMANTICS**	0.19864724	0.04288309	0.08323624
**SENSATION**	-0.0537589	0.10833767	-0.0472423
**SENSATIONS**	0.0013027	0.11805814	-0.0129654
**SENSE**	0.00876451	0	0.02791564
**SENSITIVE**	-0.0147421	-0.095146	0.03400569
**SENSITIVITY**	-0.0706342	-0.0280985	-0.168374
**SENSORY**	-0.0385823	-0.0193838	0.0955683
**SENSORY INFORMATION**	0	0.02265521	0.10225684
**SENSORY MOTOR**	0	-0.0182417	0.07249473
**SENTENCE**	0.29012245	0.05650847	-0.0339239

**SENTENCE COMPREHENSION**	0.25558574	0.11293763	0.021362
**SENTENCES**	0.28483754	0.04023049	-0.0439429
**SEQUENCE**	-0.0404405	-0.0068654	0.09006845
**SEQUENCES**	0.10921173	0.10222325	0.17256142
**SEQUENTIAL**	0.02093487	0.06872554	0.15049464
**SERIAL**	0.0352216	0.03512983	0.12771261
**SERIES**	-0.0432067	-0.0372891	-0.0057742
**SEROTONIN**	-0.0442217	-0.037616	-0.1333638
**SEVERE**	-0.105861	-0.1236113	-0.0964638
**SEVERITY**	-0.18047	-0.1726848	-0.2589758
**SEX**	-0.1290814	-0.1191823	-0.2027661
**SEXUAL**	-0.1041355	-0.0566656	-0.1078653
**SHAPE**	0.02040816	0	0.2916449
**SHAPES**	0.10425736	0	0.22721056
**SHIFT**	0.08062872	0.10425802	0.17566836
**SHIFTED**	0.06928199	0.07803686	0.05310471
**SHIFTING**	0.12853441	0.1910556	0.221261
**SHIFTS**	0.06460459	0.0786416	0.20011134
**SHORT**	0.13098561	0.01379289	0.12290508
**SHORT TERM**	0.12797212	0.08193804	0.20885394
**SIGHTED**	-0.0520638	-0.0948435	0.07725861
**SIGN**	0.13323649	0	0.11187429
**SIGNAL TASK**	0.15392649	**0.30206744**	0.05684154
**SIGNS**	0	-0.009418	0
**SILENT**	0.02268928	-0.0260873	-0.0210672
**SIMILARITY**	0.10920825	0.03157527	0.16456959
**SKILL**	0.0516958	0	0.13457731
**SKILLS**	0.03604798	-0.0299932	0.06872627
**SKIN**	-0.0547732	0.0934384	-0.1079316
**SLEEP**	-0.1711479	-0.1196185	-0.1685286
**SMOKERS**	-0.1555668	-0.0548908	-0.1031221
**SMOKING**	-0.1461889	-0.0967252	-0.1426315
**SN**	-0.089216	0.01431706	-0.1222328
**SOCIAL**	0.00889515	-0.0996002	-0.2130743
**SOCIAL COGNITION**	0.01478131	-0.0843403	-0.1117562
**SOCIAL COGNITIVE**	0	-0.0520969	-0.1081183
**SOCIAL INTERACTION**	0.0160999	-0.0415619	-0.1244231
**SOCIAL INTERACTIONS**	0.04249355	-0.0617749	-0.0849514
**SOCIALLY**	0.01448114	-0.0667366	-0.1072105
**SOLVING**	0.03939051	0.0700944	0.08473038
**SOMATOSENSORY**	-0.1187368	0	-0.0214941
**SOUND**	0.07133924	0	0.02312137
**SOUNDS**	0.03144119	-0.0544992	-0.0821471
**SPATIAL**	-0.0540038	-0.0501745	**0.37528194**

**SPATIAL ATTENTION**	0	0	**0.32127502**
**SPATIAL INFORMATION**	0	-0.0338648	0.18568346
**SPATIAL TEMPORAL**	0.03013968	0.00569255	0.04553264
**SPATIALLY**	-0.1045937	-0.1163175	0.03628692
**SPATIOTEMPORAL**	-0.0770349	-0.0958553	-0.0723357
**SPEAKER**	0.10284427	-0.0151636	-0.0489743
**SPEAKERS**	0.222386	0.08653765	0.03468429
**SPEAKING**	0.12346688	0.07218908	0
**SPECTRUM**	-0.0508527	-0.1194616	-0.1219757
**SPECTRUM DISORDER**	0.0140216	-0.0553914	-0.0803612
**SPECTRUM DISORDERS**	-0.0446545	-0.076907	-0.0993794
**SPEECH**	0.10693624	-0.0137564	-0.115902
**SPEECH PERCEPTION**	0.05267399	-0.0312051	-0.1091592
**SPEECH PRODUCTION**	0.03770663	0.00596371	-0.0524171
**SPEED**	-0.0761979	-0.0271799	0
**SPOKEN**	0.18880092	0	-0.0398807
**STATE**	-0.2717868	-0.2305219	-0.3524109
**STATIC**	0	-0.0449362	0.12994639
**STIMULI**	0.03918997	0.06536346	0.11124166
**STIMULUS**	0.10668783	0.20390408	0.2865965
**STIMULUS DRIVEN**	0.1406047	0.09733866	0.18682479
**STIMULUS RESPONSE**	0.09179423	0.17938217	0.2066013
**STOP**	0.15508636	**0.31809725**	0.0518524
**STOP SIGNAL**	0.17010304	**0.32445728**	0.06153795
**STORAGE**	0.12678604	0.1592546	0.1664466
**STRATEGIC**	0.10327214	0.12006892	0.05716827
**STRATEGIES**	0.11866015	0.13662886	0.06188153
**STRATEGY**	0.07716669	0.11486072	0.01596122
**STRESS**	-0.1900875	-0.1174818	-0.2684167
**STRESS DISORDER**	-0.1289827	-0.1061764	-0.170697
**STROKE**	-0.0862674	-0.14472	-0.1073506
**STROOP**	0.1524879	0.18540024	0.14066015
**STROOP TASK**	0.13882321	0.14482558	0.1043045
**SUBJECTIVE**	-0.1153709	-0.0042974	-0.1753879
**SUBSEQUENT MEMORY**	0	-0.0724345	-0.0400807
**SUBTRACTION**	0.11216141	0.13788763	0.28974031
**SUCCESS**	0.0816138	0.0840632	-0.018966
**SUCCESSFUL**	0.04342899	0.03786382	-0.0444783
**SUCCESSFULLY**	-0.037496	-0.0451482	0.01125643
**SUPPRESSED**	0	0.05297429	0.04330324
**SUPPRESSION**	0.03959267	0.0116006	0.0832145
**SUSTAINED**	0.01411735	0.12363113	0.07281428
**SWITCH**	0.12847421	0.173726	0.16798711
**SWITCHING**	0.15266485	0.19836746	0.2076855

**SYMBOLIC**	0.07833466	0.09563534	**0.3363543**
**SYMPTOM**	-0.1221633	-0.1137915	-0.2312992
**SYMPTOM SEVERITY**	-0.12697	-0.1130035	-0.1820895
**SYMPTOMS**	-0.202008	-0.1605709	-0.2556044
**SYNCHRONIZATION**	0	-0.0570208	-0.0219775
**SYNDROME**	-0.1940562	-0.1514387	-0.1925347
**SYNTACTIC**	**0.35118471**	0.0974278	-0.017812
**TACTILE**	-0.019656	0.00902431	0.16476398
**TAPPING**	-0.0153112	0.03515885	0.0521343
**TARGET**	0.07310734	0.12011848	0.27375804
**TARGET DETECTION**	0.05780522	0.07859634	0.08788999
**TARGETS**	0	0.05817051	0.1953787
**TASK**	0.27891988	**0.38169635**	**0.42516103**
**TASK CONDITIONS**	0.12000323	0.15001983	0.14989389
**TASK DEMANDS**	0.22028825	0.15741726	0.18167474
**TASK DIFFICULTY**	0.19424693	0.25470843	**0.31718711**
**TASK FUNCTIONAL**	0.02181292	0.06946289	0
**TASK IRRELEVANT**	0.14376408	0.11969437	0.2374225
**TASK PERFORMANCE**	0.04770857	0.15518783	0.23868872
**TASK POSITIVE**	-0.0502516	-0.0466522	0
**TASK RELEVANT**	0.11472716	0.0952952	0.23207858
**TASKS**	0.28792236	0.27516438	**0.44787769**
**TASTE**	-0.0652038	0.00583177	-0.1359218
**TEMPORALLY**	0.0628289	0.04909992	0.0278197
**TERM MEMORY**	0.15443006	0.07576216	0.2173367
**THEORY**	0.07851576	-0.019741	-0.0779424
**THEORY MIND**	0.05397523	-0.0908463	-0.1428159
**THERAPEUTIC**	-0.147515	-0.0955546	-0.1584717
**THERAPY**	-0.0727643	-0.0809771	-0.0901135
**THINKING**	0.00432478	-0.0785591	-0.0890923
**THOUGHT**	0.03132899	0.01024249	-0.0318959
**THOUGHTS**	0	-0.0241045	-0.0806277
**THREAT**	-0.0249518	0.03964256	-0.1526726
**THREATENING**	0.03119399	-0.0086262	-0.1126542
**TIME**	-0.1270053	0.02554389	0
**TIME TASK**	-0.009682	0.08222272	0.05288538
**TIMES**	0.09367623	0.1351977	0.19211926
**TIMING**	0.02340457	0.0708549	0
**TOM**	0.06360124	-0.0600445	-0.1268577
**TONE**	0.02001301	0.04411646	-0.0679547
**TONES**	0.00453131	0.02275593	-0.075184
**TOUCH**	0	0.00899349	0.12638846
**TRACK**	0	0.03089778	-0.0139003
**TRACKING**	-0.0489785	0.00304487	0.0944988

**TRAINED**	0	-0.0441719	0.15286377
**TRAINING**	-0.0503165	-0.0724065	0.04377559
**TRAIT**	-0.1056306	-0.0704592	-0.2000572
**TRAITS**	-0.039979	-0.0469464	-0.1807736
**TRAUMA**	-0.1376808	-0.1360059	-0.1843035
**TRAUMATIC**	-0.0601376	-0.0458945	-0.0808432
**TREATED**	-0.0383555	-0.0087871	-0.1091448
**TREATMENT**	-0.1841117	-0.1141892	-0.3089544
**TREATMENTS**	-0.0720405	-0.0480408	-0.1381355
**TRIAL**	0	0.1939351	0.08238302
**TRIALS**	0.15551759	0.27369564	0.2086839
**TYPICALLY DEVELOPING**	0	-0.027679	0
**UNCERTAIN**	0.04941806	0.11992615	0.09568851
**UNCERTAINTY**	0.04248017	0.15009634	0.04475735
**UNEXPECTED**	0.07710209	0.10539565	0.00391227
**UNFAMILIAR**	0.05891132	-0.0532534	0.13433144
**UNIQUE**	-0.0302538	0	-0.0454735
**UNMEDICATED**	-0.0378215	-0.0065627	-0.0768674
**UNPLEASANT**	-0.005808	0.04096639	-0.1368555
**UPDATING**	0.05799771	0.10311254	0.16053177
**VALENCE**	-0.0130674	-0.0715323	-0.1934481
**VALUABLE**	-0.0358851	-0.0046633	-0.0438239
**VALUE**	-0.1038382	-0.009991	-0.1705023
**VALUES**	-0.2202126	-0.1685159	-0.2488318
**VERB**	0.26576795	0.09895705	0
**VERBAL**	0.22165851	0.17969948	0.13630222
**VERBAL FLUENCY**	0.12204921	0.08938004	0.00460888
**VERBAL WORKING**	0.16291738	0.16287108	0.13274295
**VERBS**	0.2197505	0.03931874	0.02447462
**VIDEO**	0.03254923	-0.0482112	0.07650616
**VIDEO CLIPS**	0.06869742	-0.0406671	0.08999147
**VIDEOS**	0.01504582	-0.1254461	0.05894236
**VIEW**	-0.0021309	-0.0574232	0.07015997
**VIEWED**	-0.0211722	-0.1296996	0.01144375
**VIEWING**	-0.0435082	-0.1521619	0.04415505
**VIRTUAL**	-0.0208199	0.03268053	0.13190568
**VISION**	-0.0528482	-0.0846149	0.16799183
**VISUAL**	0	-0.1044969	**0.32414898**
**VISUAL ATTENTION**	0	-0.0209335	0.22683776
**VISUAL AUDITORY**	0.04712196	-0.0521805	0.05709513
**VISUAL FIELD**	-0.0953275	-0.1119737	0.11460871
**VISUAL INFORMATION**	0.02351922	0.00683657	0.1923056
**VISUAL MOTION**	-0.0277866	-0.0919862	0.16488856
**VISUAL PERCEPTION**	-0.0248749	-0.0871056	0.11655853

**VISUAL STIMULI**	-0.0041396	-0.0514727	0.12722814
**VISUAL STIMULUS**	-0.0067906	-0.0246719	0.09837326
**VISUAL STREAM**	-0.0165912	-0.0961425	0.11740722
**VISUAL WORD**	0.18793103	0.00716823	0.22128849
**VISUALLY**	0.06485682	0	**0.30202399**
**VISUALLY PRESENTED**	0.15459651	0.03738638	0.17061328
**VISUO**	-0.0106237	-0.0568549	0.26222165
**VISUOMOTOR**	-0.0691321	-0.0595014	0.21098076
**VISUOSPATIAL**	-0.0228223	-0.0463567	**0.32480747**
**VOCAL**	0.03035105	-0.0106807	-0.1022587
**VOICE**	0.10358112	-0.0304509	-0.0834656
**WATCHED**	0.05203201	-0.0527281	0.15652748
**WATCHING**	0	-0.0244134	0.00683524
**WM**	0.0906048	0.15230306	0.24979456
**WM TASK**	0.0683509	0.12001512	0.1909957
**WOMEN**	-0.0837248	-0.0021866	-0.1759736
**WORD**	**0.35492883**	0.11932083	0.12540783
**WORD FORM**	0.14732399	-0.0079696	0.15605229
**WORD PAIRS**	0.10064724	0.07734812	-0.0218364
**WORD RECOGNITION**	0.18227986	0.04444443	0.08865809
**WORDS**	0.29442824	0.04957296	0.05716143
**WORKING**	0.26399218	**0.32280063**	**0.4400323**
**WORKING MEMORY**	0.26835564	**0.3264132**	**0.44255216**
**WRITTEN**	0.18533492	-0.0164184	0.0639885
**YOUNG**	-0.0467842	-0.0212167	-0.0711643
**YOUNG ADULTS**	-0.0283143	-0.0126695	-0.0593087
**YOUNG HEALTHY**	-0.0421997	0.06095606	0.01246235
**YOUNGER**	0.03524195	0.03594114	0.07077296
**YOUNGER ADULTS**	0.0160155	0	0.01513588
**YOUTH**	-0.0743415	-0.0178862	-0.098062

Supplemental Table 5. NeuroSynth (Yarkoni et al., 2011) terms and their correlation with each clique. Bold = correlations > 0.30.

Eickhoff, S. B., Heim, S., Zilles, K., & Amunts, K. (2006). Testing anatomically specified hypotheses in functional imaging using cytoarchitectonic maps. *NeuroImage*, *32*(2), 570–582. https://doi.org/10.1016/j.neuroimage.2006.04.204

Eickhoff, S. B., Stephan, K. E., Mohlberg, H., Grefkes, C., Fink, G. R., Amunts, K., & Zilles, K. (2005). A new SPM toolbox for combining probabilistic cytoarchitectonic maps and functional imaging data. *NeuroImage*, *25*(4), 1325–1335. https://doi.org/10.1016/j.neuroimage.2004.12.034

Rorden, C., & Brett, M. (2000). Stereotaxic display of brain lesions. *Behavioural Neurology*, *12*(4), 191–200. https://doi.org/10.1155/2000/421719

Yarkoni, T., Poldrack, R. A., Nichols, T. E., Van Essen, D. C., & Wager, T. D. (2011). Large-scale automated synthesis of human functional neuroimaging data. *Nature Methods*, *8*(8), 665–670. https://doi.org/10.1038/nmeth.1635

Zilles, K., & Amunts, K. (2010). Centenary of Brodmann's map—Conception and fate. *Nature Reviews. Neuroscience*, *11*(2), 139–145. https://doi.org/10.1038/nrn2776
